# Benzylisoquinoline Alkaloids Biosynthesis in Sacred Lotus

**DOI:** 10.3390/molecules23112899

**Published:** 2018-11-06

**Authors:** Ivette M. Menéndez-Perdomo, Peter J. Facchini

**Affiliations:** Department of Biological Sciences, University of Calgary, Calgary, AB T2N 1N4, Canada; ivette.menendez1@ucalgary.ca

**Keywords:** benzylisoquinoline alkaloids, cytochrome P450 monooxygenase, medicinal properties, methyltransferase, *Nelumbo nucifera*, norcoclaurine synthase, sacred lotus, stereochemistry

## Abstract

Sacred lotus (*Nelumbo nucifera* Gaertn.) is an ancient aquatic plant used throughout Asia for its nutritional and medicinal properties. Benzylisoquinoline alkaloids (BIAs), mostly within the aporphine and bisbenzylisoquinoline structural categories, are among the main bioactive constituents in the plant. The alkaloids of sacred lotus exhibit promising anti-cancer, anti-arrhythmic, anti-HIV, and anti-malarial properties. Despite their pharmacological significance, BIA metabolism in this non-model plant has not been extensively investigated. In this review, we examine the diversity of BIAs in sacred lotus, with an emphasis on the distinctive stereochemistry of alkaloids found in this species. Additionally, we discuss our current understanding of the biosynthetic genes and enzymes involved in the formation of 1-benzylisoquinoline, aporphine, and bisbenzylisoquinoline alkaloids in the plant. We conclude that a comprehensive functional characterization of alkaloid biosynthetic enzymes using both in vitro and in vivo methods is required to advance our limited knowledge of BIA metabolism in the sacred lotus.

## 1. Introduction

Sacred lotus (*Nelumbo nucifera* Gaertn) is a basal eudicot aquatic plant. It belongs to the small Nelumbonaceae family (order Proteales), which includes only the genus *Nelumbo* and the species *N. lutea* (native to North America) and *N. nucifera* (native to Asia and Australia). Sacred lotus diverged from the core eudicots early in angiosperms evolutionary history, prior to a whole-genome triplication event [1,2]. According to the fossil records, Nelumbonaceae morphology has remained stable since the mid-Cretaceous period, and plastid genome sequencing analysis has established that both *Nelumbo* species, currently found in opposite sides of the Pacific Ocean, diverged relatively recently (~1.5 million years ago) from a common ancestor [3].

For thousands of years lotus has been integrated in traditional medicine, diet, and popular culture, commonly used as an ornamental plant in ponds and designated ‘sacred’ owing to its religious significance in Buddhism and Hinduism [4]. The plant also displays several outstanding characteristics rarely seen in the angiosperms, such as shoot-before-root emergence, regulated flower thermogenesis, a dim-light photosynthetic plumule, and leaves with wax-covered nanopapillae that create the self-cleaning and water-repellent ‘lotus effect’ [5,6,7]. In addition, scared lotus seeds are remarkable for their longevity (radiocarbon dated up to ~1300 years before germination), which is a feature of interest to researchers investigating the molecular mechanisms of aging resistance [8,9].

All parts of sacred lotus are medicinally significant. For example, rhizomes are used in the Chinese traditional medicine for the treatment of liver cirrhosis, dyspepsia, and dysentery; stem preparations are prescribed in Ayurvedic medicine as an anthelmintic and to treat leprosy; leaf extracts possess antiviral, diuretic, and astringent activities and are applied to fever management; flowers are employed to treat cholera and bleeding disorders; and finally seeds and dissected embryos are used as remedies for insomnia, inflammation, cancer, and heart diseases [4,10]. More than 200 secondary metabolites have been isolated from the plant and associated with various pharmacological properties [4]. The isolated compounds comprise different classes of chemicals such as flavonoids, terpenoids, and alkaloids. In particular, benzylisoquinoline alkaloids (BIAs) in the aporphine and bisbenzylisoquinoline structural categories are among the main bioactive constituents of sacred lotus. 

Nuciferine, the major aporphine found in *N. nucifera*, has reportedly been effective in the treatment of several types of cancer (e.g., lung, colon, and breast) and neuroblastoma [11,12,13]. Other bioactive aporphines extracted from sacred lotus are pronuciferine, an alkaloid reported to decrease intracellular triglyceride content in adipocytes, making it a promising metabolite to treat obesity [14]; *N*-nornuciferine, which acts as a melanogenesis inhibitor [15,16]; *O*-nornuciferine (also known as *N*-methylasimilobine) and lysicamine, both possessing potent antioxidant properties; 7-hydroxydehydronuciferine, a metabolite that significantly inhibits the proliferation of melanoma, prostate, and gastric cancer cells [17]; and roemerine, which has been ascribed anti-fungal and anti-malarial properties [18]. 

Neferine and liensinine are the main bisbenzylisoquinoline components of sacred lotus. The former has been reported to possess anti-arrhythmic effects [19] and to induce apoptosis in human lung cancer [20]; hepatocellular carcinoma [21], and ovarian cancer [22], whereas the latter has been shown to inhibit the growth of breast cancer cells and prevent associated bone destruction [12,23]. In addition, neferine, liensinine, isoliensinine, and nelumboferine exhibit sedative effects in mice models, which could be related to the relaxation properties of tea prepared from sacred lotus embryos [24]. 

Despite the medicinal importance of BIAs in sacred lotus, relatively little is known about alkaloid metabolic pathways in the plant, or the biosynthetic genes and cognate enzymes. Remarkably, although many BIAs isolated from *N. nucifera* have been detected in the *R*-enantiomer conformation, which contrasts with predominantly *S*-enantiomer conformers found in opium poppy (*Papaver somniferum*) and related plants (order Ranunculales), no studies have yet examined this feature of BIA metabolism in sacred lotus. Such topics are of significance to advance research in the field of plant alkaloid metabolism, particularly in non-model species, and to develop better strategies to bioengineer high-value phytopharmaceuticals in various production systems. Herein, we review the current knowledge of BIAs biosynthesis in sacred lotus.

## 2. Occurrence of BIAs in Sacred Lotus

Benzylisoquinoline alkaloids (BIAs) constitute a large class of plant specialized metabolites derived from tyrosine [25]. The enantioselective condensation of dopamine (yielding the tetrahydroisoquinoline moiety) and 4-hydroxyphenylacetaldehyde (yielding the benzyl moiety) by norcoclaurine synthase (NCS) generates (*S*)-norcoclaurine, the common precursor to all BIAs [26]. A variety of coupling reactions and functional group modifications establish a large number of structural subcategories and specific compounds with a plethora of pharmacological properties. These include the narcotic analgesics morphine and codeine (morphinans), the muscle relaxant papaverine (1-benzylisoquinoline), the antimicrobial agents sanguinarine (benzophenanthridine) and berberine (protoberberine), the bronchodilator and anti-inflammatory glaucine (aporphine), and the potential anticancer drugs noscapine (phthalideisoquinoline) and dauricine (bisbenzylisoquinoline) [27]. 

The occurrence of BIAs in nature is restricted to certain plant families, primarily in the order Ranunculales. Consequently, most of the plants that have been investigated with respect to BIA metabolism are members of the Papaveraceae, Ranunculaceae, Berberidaceae, and Menispermaceae families [27,28,29,30]. The first and foremost of these is opium poppy (*Papaver somniferum*). However, BIAs also occur sporadically in the orders Piperales and Magnoliales, as well as in the Rutaceae, Lauraceae, Cornaceae and Nelumbonaceae families [31], but plants belonging to these taxa have received far less attention. Sacred lotus leaves, embryos, and leaf sap are rich in BIAs, although the alkaloid composition and content varies considerably among the nearly 600 known genotypes [32,33,34,35]. There are three main subclasses of BIAs in sacred lotus: the 1-benzylisoquinoline, aporphine, and bisbenzylisoquinoline alkaloids (Table 1).

### 2.1. 1-Benzylisoquinoline Alkaloids

1-Benzylisoquinoline alkaloids occur in trace amounts in several sacred lotus organs (Table 1). Norcoclaurine, also known as higenamine, is the common intermediate for the biosynthesis of all BIAs. It was first reported in sacred lotus embryos a half-century ago [36] and later isolated from *N. nucifera* leaves via anti-HIV bioassay-guided fractionation [37]. Norcoclaurine is notable for its anti-inflammatory, anti-arrhythmic, and anti-thrombotic properties, and is also considered a β-adrenergic receptor agonist [47]. 

**Figure 1:**
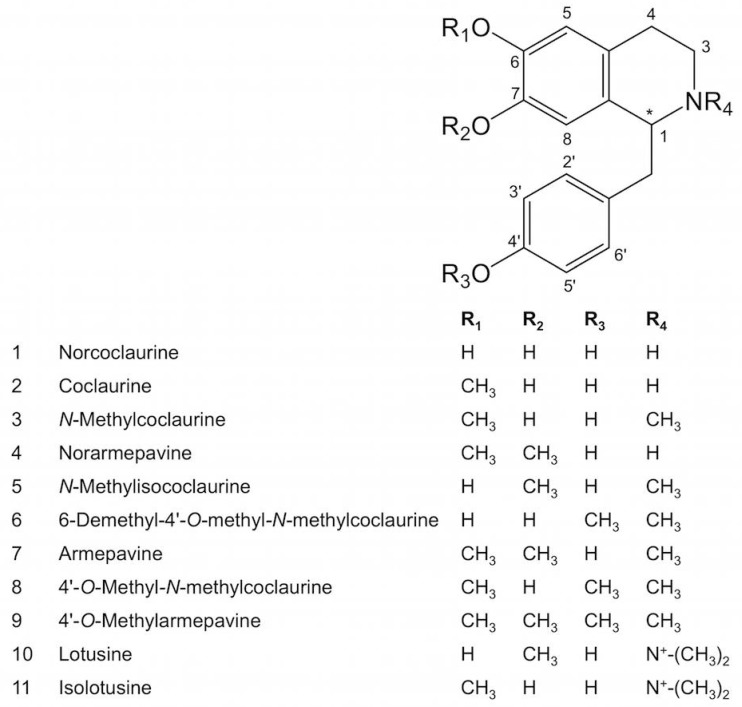
Major 1-benzylisoquinoline alkaloids reported in sacred lotus. Asterisk indicates a chiral center.

Norcoclaurine subsequently undergoes *O*- and *N*-methylation yielding various 1-benzylisoquinoline alkaloid derivatives (Figure 1). Coclaurine (6-*O*-methylated norcoclaurine), norarmepavine (7-*O*-methylated coclaurine), *N*-methylcoclaurine (*N*-methylated coclaurine), and armepavine (7-*O*- and *N*-methylated coclaurine) isolated from sacred lotus flowers have shown melanogenesis inhibition activity, with potential application in the cosmetics industry [15]. Armepavine also exhibited a suppression of T-cell proliferation and an inactivation of NF-kB, among other immunomodulatory effects that could be beneficial for the treatment of autoimmune diseases such as systemic lupus erythematosus and crescentic glomerulonephritis [42,48]. Two quaternary amines, lotusine and isolotusine (most likely derived from *N*-methylisococlaurine and *N*-methylcoclaurine, respectively) and three 4′-methoxylated compounds, 6-demethyl-4′-*O*-methyl-*N*-methylcoclaurine, 4′-*O*-methyl-*N*-methylcoclaurine, and 4′-*O*-methylarmepavine have also been isolated from the plant [38,43].

### 2.2. Aporphines

**Figure 2:**
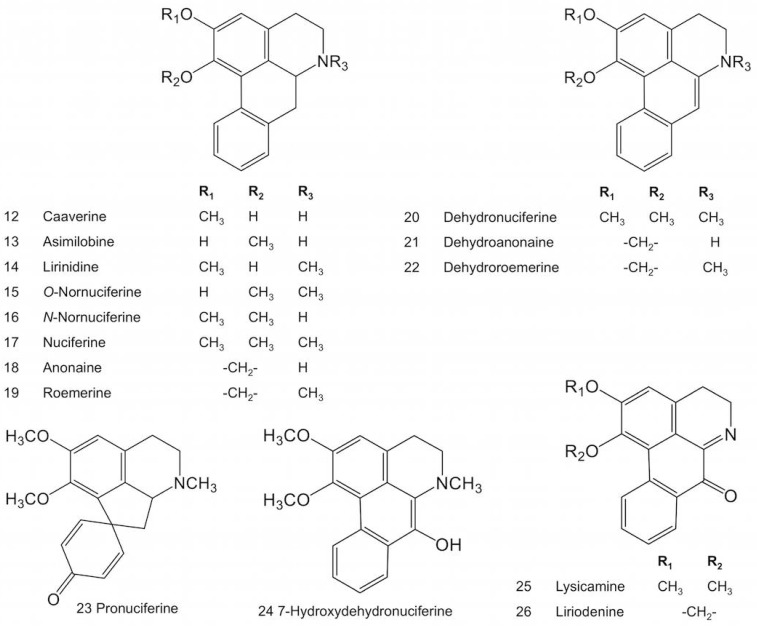
Major aporphine alkaloids reported in sacred lotus.

*Nelumbo nucifera* contains several aporphine alkaloids that accumulate mainly in the leaves (Table 1). Nuciferine is the major alkaloid in this organ, although substantial variation in alkaloid content and composition is found between cultivars [32,33,35,49]. Aporphines in sacred lotus (Figure 2) are presumably derived from 1-benzylisoquinoline intermediates as a result of C8-C2′ coupling reactions, except for pronuciferine, which exhibits C8-C1′ coupling. The C6 and/or C7 positions (Figure 1) are consistently *O*-methylated, with the only exceptions being anonaine and roemerine (and their dehydro derivatives), in which a methylenedioxy bridge occurs between these carbon atoms. The isoquinoline ring can also be *N*-methylated, but there are no reports of quaternary (e.g., *N*,*N*-dimethylated) amines among sacred lotus aporphines. 

Curiously, all aporphines reported in *N. nucifera* lack of substitutions in the benzyl moiety presumably derived from 4-hydroxyphenylacetaldehyde. This is a major difference with respect to the aporphines isolated from members of the Ranunculales, such as corytuberine, magnoflorine, isoboldine, and glaucine that are all likely derived via reticuline [50,51]. Reticuline is a 1-benzylisoquinoline alkaloid containing 3′-hydroxyl and a 4′-methoxyl groups and serving as a key branch point intermediate in the biosynthesis of most BIAs in the Ranunculales [27]. However, reticuline has not been reported in sacred lotus, and its absence could indicate the existence of substantial differences in BIA metabolism in this ancient plant. Nevertheless, all 1-benzylisoquinolines isolated from *N. nucifera* so far display a 4′-hydroxyl or 4′-methoxyl moiety, and it remains unclear why aporphines in the plant lack these modifications. Further research on aporphine biosynthesis is required to shed light on this intriguing phenomenon.

### 2.3. Bisbenzylisoquinolines

Bisbenzylisoquinoline alkaloids accumulate predominantly in the seed embryo of *N. nucifera*. Although it has been suggested that these alkaloids are synthesized in the leaf and then transported in the leaf sap to the embryo [32], the localization of BIA biosynthesis has not been experimentally examined. Neferine and liensinine are the major alkaloid constituents of the embryo, although the bisbenzylisoquinolines profile varies considerably in different genotypes [32,35].

**Figure 3:**
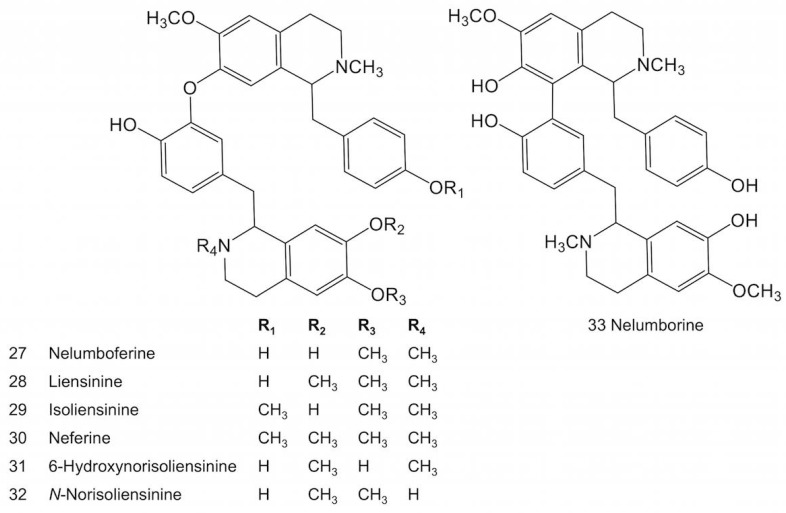
Major bisbenzylisoquinoline alkaloids reported in sacred lotus.

**Figure 4:**
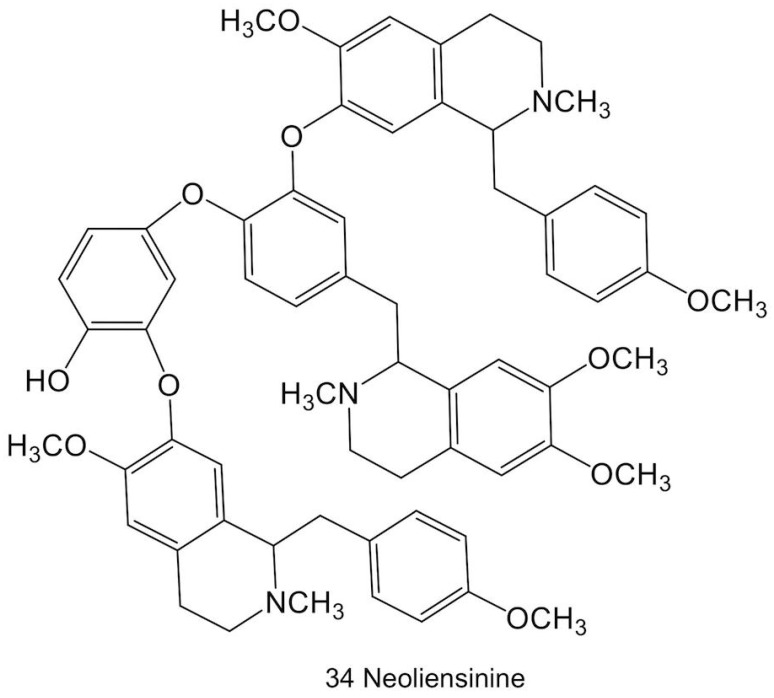
The tribenzylisoquinoline alkaloid, neoliensinine, reported in sacred lotus.

Bisbenzylisoquinoline alkaloids are formed by C6-*O*-C3′ or C8-C3′ coupling (or C8-C5′ coupling, due to free rotation of the benzyl ring) between two 1-benzylisoquinoline monomers. Different *O*- and *N*-methylation patterns create a diverse array of compounds (Figure 3). A more complex structure, the tribenzylisoquinoline alkaloid neoliensinine (Figure 4), has been recently isolated from sacred lotus embryos [46]. As with aporphine biosynthesis, it is not yet known whether the differential *O*- and *N*-methylation patterns among bisbenzylisoquinolines and tribenzylisoquinolines are established before or after coupling.

## 3. Stereochemistry of BIAs Biosynthesis in *Nelumbo nucifera*

In members of the Ranunculales, BIA stereochemistry is initially introduced by the enantioselective Pictet-Spengler condensation of dopamine and 4-hydroxyphenylacetaldehyde catalyzed by norcoclaurine synthase (NCS) [26]. Norcoclaurine contains a stereogenic atom and can theoretically occur as either the *R* or *S* conformer. To date, all functionally characterized NCS from opium poppy and related members of the Ranunculales yield exclusively (*S*)-norcoclaurine [52,53,54,55,56,57,58,59]. In contrast, norcoclaurine was first reported as an *R*-enantiomer in sacred lotus [36], a result later confirmed by HPLC coupled with chiral fluorescent detection [39]. The *S*-enantiomer of norcoclaurine has also been reported from *N. nucifera* leaves [37] (Table 1). In addition, it was recently reported that the embryos contained a racemic mix (59:41) of norcoclaurine-4′-*O*-β-d-glucoside 1*R* and 1*S* diastereomers [60], further supporting the formation of both *R* and *S* norcoclaurine conformers. 

At least five functional isoforms of norcoclaurine synthase (NnNCS) have been purported in sacred lotus, based on the occurrence of homologs in the genome [34], and in various transcriptomes [34,49,61,62]. These NCS candidates must be functionally characterized to determine which, if any, are responsible for the formation of norcoclaurine and variations in the stereochemistry of BIAs in sacred lotus. Since norcoclaurine has been isolated in both *R* and *S* conformations, it is possible that two or more NnNCS isoforms catalyze the Pictet-Spengler condensation with the opposite enantioselectivity. Alternatively, it is also plausible that the diverse BIA stereochemistry in sacred lotus results from a lack of NCS enantioselectivity, leading to the formation of both enantiomers by one enzyme. The unique features of the BIA biosynthetic enzymes from sacred lotus provide exciting targets for advanced structural and biochemical investigations with respect to substrate recognition, product formation and underlying catalytic mechanisms.

Optically active enantiomers or stereoisomers exhibiting similar physicochemical properties might differ in their biological activity. Although the specific physiological role of norcoclaurine is not known, the two enantiomers could be associated with differences in substrate recognition by NCS, and in other biochemical interactions. For example, (*S*)-norcoclaurine appears superior to (*R*)-norcoclaurine in suppressing the inducible expression of nitric oxide synthase, a hallmark of septic shock [63]. 

The majority of BIAs isolated from sacred lotus are *R*-conformers (Table 1). In contrast, most BIAs in members of the Ranunculales are *S*-conformers, with the notable exception of the bisbenzylisoquinolines (1*R*,1′*S*)-berbamunine, (1*R*,1′*S*)-2′-norberbamunine, and (1*R*,1′*R*)-guatteguamerine from *Berberis stolonifera* (presumably derived from (*R*)- and (*S*)-coclaurine and/or (*R*)- and (*S*)-*N*-methylcoclaurine) [64]. Although aporphines in the *S* conformation have not been reported in sacred lotus (except for racemic pronuciferine [43], a proaporphine), some 1-benzylisoquinoline alkaloids, such as norcoclaurine and armepavine, have been detected as both *R* and *S* enantiomers [16,36,37,39,40,48]. Moreover, the bisbenzylisoquinoline and tribenzylisoquinoline alkaloids have been reported as combinations of various *R* and *S* 1-benzylisoquinolines with the exception of (1*R*,1′*R*)-liensinine [46]. 

The stereospecificity (i.e., the ability to distinguish between stereoisomers) of downstream BIA biosynthetic enzymes is not known. For example, norcoclaurine-6-*O*-methyltransferase from *Thalictrum flavum* accepts both (*R*)- and (*S*)-norlaudanosoline (an unnatural analogue of norcoclaurine) [65]. In opium poppy, (*S*)-reticuline is converted by (i) the berberine bridge enzyme to (*S*)-scoulerine to form benzophenanthridine, protoberberine, and/or phthalideisoquinoline alkaloids and; (ii) corytuberine synthase to (*S*)-corytuberine leading to aporphine alkaloids. In contrast, reticuline epimerase is the gateway enzyme in the conversion of (*R*)-reticuline to morphinan alkaloids since the subsequent enzyme salutaridine synthase (CYP719B1) does not accept (*S*)-reticuline [30,66]. Enzyme stereospecificity is a critical feature of BIA metabolism in the Ranunculales. The stereospecificity of BIA biosynthetic enzymes in sacred lotus might be considerably different based on the widespread occurrence of *R*-conformers.

## 4. BIA biosynthetic Genes and Enzymes in the Sacred Lotus

**Figure 5:**
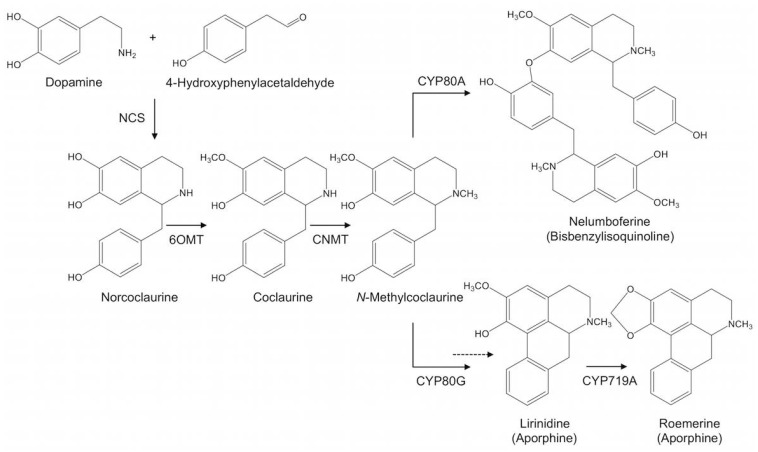
Suggested BIA biosynthetic pathway in sacred lotus. The scheme shows norcoclaurine as the common precursor and *N*-methylcoclaurine as the branch point intermediate in the formation of aporphine and bisbenzylisoquinoline alkaloids. Tailoring reactions, such as *O*- and *N*-methylations, hydroxylation, oxidation, C-C and C-O coupling (and a possible dehydration represented by the dashed arrow) yield the diverse BIAs reported in *Nelumbo nucifera*. Note that other 1-benzylisoquinolines derived from norcoclaurine could be used in the formation of aporphine and bisbenzylisoquinoline alkaloids, as this representation is merely one of many possible routes (e.g., major BIAs in sacred lotus such as nuciferine and neferine are not represented). Stereochemistry has been omitted for simplicity. Abbreviations: 6OMT, norcoclaurine 6-*O*-methyltransferase; CNMT, coclaurine *N*-methyltransferase; CYP719A, cytochrome P450 monooxygenase 719A, CYP80A, cytochrome P450 monooxygenase 80A; CYP80G: cytochrome P450 monooxygenase 80G; NCS, norcoclaurine synthase.

Analysis of the *Nelumbo nucifera* genome sequence suggest that, after the lineage-specific whole-genome duplication event (approximately 18 to 76 million years ago), several rearrangements (i.e., ancestral chromosome fissions, fusions, and a single inversion) were responsible for the modern diploid karyotype (16 chromosomes) [67,68]. The sacred lotus genome is ~1 Gb in size and encodes approximately 27,000 genes [1,2]. Based on sequence similarity with BIA biosynthetic genes in members of the Ranunculales [30], genes predicted to encode norcoclaurine synthase (NCS), *O*- and *N*-methyltransferases (OMT and NMT), and cytochrome P450 (CYP) monooxygenases CYP80A, CYP80G, and CYP719A (CYP) have been detected in the sacred lotus genome (Figure 5). Although other gene candidates have been suggested to participate in morphinan and protoberberine alkaloid biosynthesis [49], compounds belonging to these structural groups have not been detected in sacred lotus. Herein, we discuss only enzymes potentially involved in 1-benzylisoquinoline, aporphine and bisbenzylisoquinoline alkaloid pathways (Table 2). However, functional characterization of these enzymes has not been investigated. Most work has focused on the aporphine metabolism, primarily through correlational analysis of gene expression profiles and alkaloid content in different organs and developmental stages of sacred lotus [34,49,61,62,69]. 

### 4.1. Norcoclaurine Synthase

NCS catalyzes the condensation of dopamine and 4-hydroxyphenylacetaldehyde as the first committed step in BIA metabolism (Figure 5). In members of the Ranunculales, NCS belongs to the PR10/Bet-v1 family of proteins [54,59]. NCS activity has been detected in crude protein extracts of sacred lotus leaves, but not in petioles or roots [31]. Seven genes putatively encoding NCS have been described in the sacred lotus genome [34]. All of these genes contained two exons separated by one intron. *NnNCS1*, *NnNCS4*, and *NnNCS5* were clustered together along with two pseudogenes (*NnNCS2* and *NnNCS6*). *NnNCS3* and *NnNCS7* were not on the same genomic scaffold, but could also be part of the gene cluster. All predicted proteins contained a canonical glycine-rich loop and conserved catalytic residues (K-122 and E-110) described in the functionally and structurally characterized NCS from *T. flavum* (TfNCS) [52,56]. 

Correlational analysis of genes expression profiles and alkaloid content were interpreted to suggest that only NnNCS7 plays a major role in BIA biosynthesis [34]. However, even though the *NnNCS7* gene was predominantly expressed in all tested organs and developmental stages, significant variation in transcript level was detected depending on the cultivar analyzed. For example, only *NnNCS7* was (highly) expressed in mature leaves of the Xuehuou variety, although the alkaloid content was the lowest among 10 tested cultivars. In contrast, despite a low level of *NnNCS7* expression in mature leaves of the WSL40 cultivar, the alkaloid content was more than double that detected in the leaves of Xuehuou [34]. In addition, expression of *NnNCS7* has been reported to increase with leaf development, with peak levels between the folded and unfolded stages, which is prior to the detected accumulation of alkaloids [61]. However, *NnNCS7* gene expression was higher in the low-alkaloid cultivar Luming compared with the high-alkaloid cultivar WD40 [61]. Similar inconsistencies have been detected depending on the plant organ and developmental stage analyzed [34]. Inconsistencies between gene expression profiles and alkaloid content were suggested to result from the possibility that NCS in sacred lotus is a functional heterodimer composed of cultivar-dependent isoforms [34]. It is notable that TfNCS has been structurally and functionally characterized as a homodimer [52,57]. Functional characterization of putative NCS isoforms from sacred lotus is required to support their role in BIA metabolism. It is also notable that at least five putative tyrosine decarboxylase (*TYDC*) genes are expressed in lotus leaves [61]. TYDC catalyzes the decarboxylation of tyrosine and L-DOPA yielding, indirectly or directly, the NCS substrate dopamine. Putative TYDC enzymes also require functional characterization.

### 4.2. Methyltransferases

Norcoclaurine is modified by a series of *O*- and *N*-methylations to form the diverse 1-benzylisoquinolines reported in sacred lotus (Figure 1). Both *O*-methyltransferases (OMTs) and *N*-methyltransferases (NMTs) use S-adenosyl-L-methionine as the methyl donor [70]. Norcoclaurine contains three hydroxyl groups at C6, C7, and C4′ and a secondary nitrogen in the isoquinoline ring, all of which are susceptible to methylation. 

In the Ranunculales, the 6-*O*-methylation of norcoclaurine to coclaurine is the first tailoring reaction in BIAs biosynthesis [30] and it is expected that a similar reaction occurs in sacred lotus (Figure 5). Based on sequence similarity with OMTs from members of the Ranunculales, four candidate norcoclaurine-6-*O*-methyltransferase (*Nn6OMT*) genes have been detected in the sacred lotus genome, with two of the genes (*Nn6OMT2* and *Nn6OMT3*) clustered [49,61,62]. *Nn6OMT1* showed the highest expression levels in leaves, with a peak at early stages of development, and cultivar-dependent expression aligned with cultivar-specific alkaloid content [49,61]. In addition, the *Nn6OMT1* isoform also contains conserved catalytic residues (H-256, D-257, and E-315) described in *T. flavum* 6OMT [65].

Several 1-benzylisoquinolines from sacred lotus, including norarmepavine, armepavine, and major aporphines and bisbenzylisoquinolines are *O*-methylated at C7; thus, enzymes capable of C7-*O*-methylation are also expected to occur. Five candidate genes putatively encoding 7-*O*-methyltransferases (*Nn7OMTs*) have been detected in the *N. nucifera* genome based on sequence similarity with OMTs from members of the Ranunculales. However, only *Nn7OMT1-3* genes have shown significant expression in the leaves [61]. The other two candidates *Nn7OMT4* and *Nn7OMT5* were previously proposed as *Nn4*′*OMT1* and *Nn4*′*OMT4*, respectively, along with two other genes encoding putative 4′-*O*-methyltransferases (*Nn4*′*OMTs*) [49]. The expression levels of the corresponding *Nn4’OMT* genes in the leaves of two cultivars were low or conflicted with the cultivar-specific alkaloid content, except for *Nn4*′*OMT1* [49]. Owing to the isolation of several 1-benzylisoquinoline and bisbenzylisoquinoline alkaloids containing a 4′-methoxy group, an enzyme associated with 4′OMT activity should occur in the plant. It is possible that OMTs in sacred lotus lack strict regiospecificity and enzymes that primarily function as a 6OMT or a 7OMT also catalyze 4′-*O*-methylation on certain substrates. Alternatively, the occurrence of OMT heterodimers that perform distinct *O*-methylations has recently been described in opium poppy [71]. Similarly, certain O-methylation activities could be associated with the possible formation of OMT heterodimers in sacred lotus.

Only a single gene candidate putatively encoding coclaurine *N*-methyltransferase (*NnCNMT1*) has been detected in the sacred lotus genome [49,61]; however, a recent report identified two additional candidates, one of which (*NnCNMT2*) is clearly a pseudogene [62]. *NnCNMT1* expression in sacred lotus leaves increased progressively with developmental stage and alkaloid content, although expression levels were 10-fold lower than *Nn6OMT1*, the preceding enzyme in the biosynthetic pathway [49]. In addition, *NnCNMTs* expression was substantially different among sacred lotus cultivars. In some cases *NnCNMTs* transcripts were undetectable in cultivars such as Bua Khem Chin1200 with a high alkaloid content [62]. CNMT catalytic activity is a critical step in sacred lotus BIA metabolism owing to the putative role of *N*-methylcoclaurine as a key branch point intermediate in the formation of aporphines and bisbenzylisoquinolines [61]. It is also possible that aporphine and bisbenzylisoquinoline, rather than 1-benzylisoquinoline intermediates, are themselves *O*- and/or *N*-methylated.

### 4.3. Cytochrome P450 Monooxygenases

Cytochrome P450 monooxygenases (CYPs) constitute a large group of heme proteins catalyzing diverse reactions in plant specialized metabolism. The enzymes are activated by the transfer of two electrons from NADPH via a NADPH-cytochrome P450 reductase [72]. Two main CYP families are proposed to play a key role in BIA biosynthesis in sacred lotus: CYP80 (subfamilies A and G) and CYP719A [69].

The CYP80A subfamily has been associated with bisbenzylisoquinoline alkaloids biosynthesis in the Ranunculales. For example, the enzyme CYP80A1 (berbamunine synthase) isolated from *Berberis stolonifera* catalyzes C-O phenol coupling of 1-benzylisoquinoline substrates to form (1*R*,1’*S*)-berbamunine and other dimeric BIAs [64]. In lotus genome, only one *NnCYP80A* candidate has been detected, and its expression was positively correlated with alkaloid content [61]. Curiously, the gene showed differential spatial expression, with high levels in the embryo and significantly lower levels in the leaves. In a previous study based on phylogenetic analysis, this gene was proposed as a second CYP80G isoform [49], demonstrating the need for proper functional characterization of the corresponding enzymes.

The CYP80G subfamily has been correlated with aporphine alkaloid biosynthesis. In *Coptis japonica*, CYP80G2 (corytuberine synthase) catalyzes the conversion of (*S*)-reticuline to (*S*)-corytuberine via intramolecular C-C coupling [73]. However, neither reticuline nor corytuberine have been isolated from sacred lotus; thus, other 1-benzylisoquinoline intermediates are likely involved in aporphine alkaloid biosynthesis. Transcripts of CYP80G homologs in sacred lotus leaves were detected using digital gene expression analysis and according to the observed expression pattern only one was proposed to be implicated in the aporphine biosynthesis [49]. In a recent study, the expression of this *NnCYP80G* gene was reported at high levels in leaves and transcript levels and aporphine alkaloid content were induced after mechanical wounding [62]. Likewise, the expression profile of *NnCYP80G* showed high transcript levels in the leaves but significantly lower levels in the embryos, opposite to what was observed for *NnCYP80A* [61]. Interestingly, *NnCYP80A* and *NnCYP80G* are clustered within a 20 kb region in the *N. nucifera* genome, suggesting functional divergence after duplication [61].

Members of the CYP719A subfamily typically catalyze methylenedioxy bridge formation in the Ranunculales leading to the formation of (*S*)-stylopine (CYP719A20), (*S*)-canadine (CYP719A21), and (*S*)-cheilanthifoline (CYP719A25) [74]. In sacred lotus, aporphines such as anonaine and roemerine (and their dehydro derivatives) contain a methylenedioxy bridge. Interestingly, only the *NnCYP719A22* gene from sacred lotus has been suggested to function in alkaloid biosynthesis [69] (Figure 5).

At least two transcript candidates encoding *N*-methylcoclaurine 3′-hydroxylase (NMCH; CYP80B subfamily) have been detected in sacred lotus leaves [49]. NMCH is involved in the hydroxylation of *N*-methylcoclaurine, which is required for reticuline biosynthesis in the Ranunculales [27]. However, as reticuline has not been detected in sacred lotus and 3′-hydroxylation is not a feature of any reported alkaloids from the plant, a functional NMCH homolog is unlikely to occur.

### 4.4. Other Enzymes

Expression of genes involved in the formation of morphinan (codeine 3-*O*-demethylase, CODM, and thebaine 6-*O*-demethylase, T6ODM), and protoberberine (scoulerine-9-*O*-methyltransferase, SOMT) alkaloids has also been considered in sacred lotus [49]. However, morphinan and protoberberine alkaloids have not been detected in *N. nucifera* [4,10]; thus, it is questionable whether these enzyme candidates are involved in BIA biosynthesis. However, it has been suggested that *O*- and *N*-demethylases could participate in tailoring reactions in aporphine biosynthesis [61]. 

The biosynthesis of aporphine alkaloids in sacred lotus could involve a dehydration reaction to remove the 4’-hydroxyl group from a 1-benzylisoquinoline or aporphine intermediate. Comparing the gene expression profiles of sacred lotus cultivars with markedly different aporphine profiles [33] could facilitate the detection of additional missing or unanticipated enzymes in BIA metabolism. 

### 4.5. Functional Characterization

The elucidation of BIA biosynthetic pathways requires a thorough biochemical and physiological characterization of relevant enzymes. To date, no BIA biosynthetic enzymes from sacred lotus have been functionally analyzed. In vitro experiments using purified proteins are generally the first step in the functional characterization of enzyme candidates. Alternatively, enzyme function can be evaluated in vivo using engineered bacterial (e.g., *Escherichia coli*) and yeast (e.g., *Saccharomyces cerevisiae*) systems [51,55]. Immunoprecipitation have been used to support the physiological significance of NCS in the formation of (*S*)-norcoclaurine in opium poppy [54]. In planta techniques, such as candidate gene overexpression and RNA interference [75,76,77], and gene knockout using CRISPR/Cas9 technology [78] have also been used to demonstrate physiological relevance. Virus-induced gene silencing (VIGS) has been effective to assess the impact of candidate gene suppression on BIA biosynthesis in opium poppy [50,79,80]. Similar approaches must be developed to advance the functional characterization of biosynthetic genes and enzymes in sacred lotus.

### 4.6. Regulation and Localization of BIA Biosynthesis in Sacred Lotus

BIA metabolism in sacred lotus appears tightly regulated, with different organs showing specific alkaloids profiles (e.g., aporphines and bisbenzylisoquinoline accumulate in the leaves and embryos, respectively). In members of the Ranunculales, at least two transcription factors (TFs) have been implicated in the regulation of BIA metabolism: WRKY (*CjWRKY1*) and bHLH1 (*CjbHLH1*) [81,82]. MYB family TFs have been proposed to play a major role in regulating alkaloid biosynthesis in sacred lotus leaves [61]. This conclusion was based on correlations between the expression levels of putative transcription factor genes and selected genes putatively encoding biosynthetic enzymes, such as TYDC, NCS, CNMT, and CYP80G as well as TF-promoter interactions for *NnMYB6*, *NnMYB12*, and *NnMYB113* evaluated using dual luciferase assays [61]. Such deductions are compromised by the lack of functional data supporting the biochemical and physiological roles of these enzyme candidates. In addition, some variants of *NnWRKY* and *NnbHLH1* were not linked to BIA biosynthetic gene expression, although high expression levels of *NnWRKY* were detected [61]. 

Another aspect of BIA metabolism in sacred lotus that has received little attention is the cellular and subcellular localization of alkaloid biosynthesis. Cytosolic localization of NCS was suggested based on the absence of signal peptides on the candidate enzymes [34]. In addition, putative NCS transcripts are primarily found in leaves [34], suggesting this organ as a major site of BIA biosynthesis. If validated, this is markedly different from the abundance of NCS in the rhizome and root in *T*. *flavum* [59] and opium poppy [54], respectively. 

In opium poppy, most BIA biosynthetic genes are expressed in companion cells, and the cognate biosynthetic enzymes are associated with sieve elements of the phloem. The final stages of BIA biosynthesis, and the ultimate storage of alkaloids occurs in specialized laticifers [83]. The rhizome, leaf, petiole, and peduncle of sacred lotus also contain laticifers associated with vascular bundles, mainly in the parenchyma between the phloem and xylem [84]. However, the roles of sacred lotus laticifers in BIA metabolism and storage are not known. Immunoblot and real-time PCR analyses using total protein and RNA extracts, respectively, from various organs of sacred lotus will provide valuable information on the localization of validated biosynthetic enzymes and corresponding gene transcripts [54]. The cell-type specific occurrence of enzymes and cognate transcripts can then be determined by immunofluorescence labeling and in situ RNA hybridization, respectively [85].

## 5. Conclusions

BIAs constitute a substantial part of humankind’s traditional and modern medicine [27]. A vast number of BIA biosynthetic genes and enzymes have been isolated from members of the Ranunculales, especially from opium poppy [86]. The ancient aquatic plant sacred lotus has long been exploited for its medicinal properties, which are largely conferred by aporphine and bisbenzylisoquinoline alkaloids. Interestingly, most BIAs found in sacred lotus are *R*-conformers [16,17,37,39,40,46], contrary to the prevalence of *S*-conformers in the Ranunculales. Therefore, the unusual stereochemistry of alkaloids in this basal eudicot is worthy of research at the molecular and biochemical levels. The availability of a draft sacred lotus genome sequence underpins opportunities to isolate BIA biosynthetic genes and enzymes. However, a definitive elucidation of biosynthetic pathways requires thorough biochemical and physiological characterization of putative genes and enzymes.

## Figures and Tables

**Table 1:** Benzylisoquinoline alkaloids (BIAs) detected in different organs of *Nelumbo nucifera*, their chemical formula, and stereochemistry. Alkaloid structures are assigned numbers as shown in Figure 1, Figure 2, Figure 3 and Figure 4. L: leaf, E: embryo, F: flower, S: seed, R: rhizome, LS: leaf sap, NS: not specified, N/A: not applicable.

No.	Alkaloid	Formula	Enantiomer	Organ	Reference
**1-BENZYLISOQUINOLINE**					
1	Norcoclaurine	C_16_H_17_NO_3_	(+)-*R* and (−)-*S*	L, E	[36,37,38,39]
2	Coclaurine	C_17_H_19_NO_3_	(+)-*R*	L, E, F	[15,37,38]
3	*N*-Methylcoclaurine	C_18_H_21_NO_3_	(−)-*R*	L, E, F	[15,37,38]
4	Norarmepavine	C_18_H_21_NO_3_	(+)-*R*	F	[15,40]
5	*N*-Methylisococlaurine	C_18_H_21_NO_3_	NS	L, E	[38,41]
6	6-Demethyl-4′-*O*-methyl*-N*-methylcoclaurine	C_18_H_21_NO_3_	NS	E	[38]
7	Armepavine	C_19_H_23_NO_3_	(−)-*R* and (+)-*S*	L, E, S	[15,38,40,42]
8	4′-*O*-Methyl*-N*-methylcoclaurine	C_19_H_23_NO_3_	NS	E	[38]
9	4′-*O*-Methylarmepavine	C_20_H_25_NO_3_	NS	L	[43]
10	Lotusine	C_19_H_24_NO_3_^+^	NS	E	[38]
11	Isolotusine	C_19_H_24_NO_3_^+^	NS	E	[38]
**APORPHINE**					
12	Caaverine	C_17_H_17_NO_2_	(−)-*R*	L	[17,40]
13	Asimilobine	C_17_H_17_NO_2_	(−)-*R*	L, F	[15,17,44]
14	Lirinidine	C_18_H_19_NO_2_	(−)-*R*	L, F	[16]
15	*O*-Nornuciferine	C_18_H_19_NO_2_	(−)-*R*	L, F	[16,17,35]
16	*N*-Nornuciferine	C_18_H_19_NO_2_	(−)-*R*	L, E, F	[16,17,38]
17	Nuciferine	C_19_H_21_NO_2_	(−)-*R*	L, E, F	[15,17,35,38]
18	Anonaine	C_17_H_15_NO_2_	(−)-*R*	L, F	[17,32]
19	Roemerine	C_18_H_17_NO_2_	(−)-*R*	L, F	[17,18,32,35]
20	Dehydronuciferine	C_19_H_19_NO_2_	N/A	L, R	[16,32,41]
21	Dehydroanonaine	C_17_H_13_NO_2_	N/A	L	[41]
22	Dehydroroemerine	C_18_H_15_NO_2_	N/A	L	[41]
23	Pronuciferine	C_19_H_21_NO_3_	(+)-*R* and (−)-*S*	L, E, F	[16,38,40,43]
24	7-Hydroxydehydronuciferine	C_19_H_19_NO_3_	N/A	L	[17]
25	Lysicamine	C_18_H_13_NO_3_	N/A	L, F	[16]
26	Liriodenine	C_17_H_9_NO_3_	N/A	L	[17]
**BISBENZYLISOQUINOLINE**					
27	Nelumboferine	C_36_H_40_N_2_O_6_	NS	E, LS	[32,45]
28	Liensinine	C_37_H_42_N_2_O_6_	1*R*,1′*R*	L, E, F, LS	[32,35,44,46]
29	Isoliensinine	C_37_H_42_N_2_O_6_	1*R*,1′*S*	E	[35,46]
30	Neferine	C_38_H_44_N_2_O_6_	1*R*,1′*S*	E, LS	[32,35,46]
31	6-Hydroxynorisoliensinine	C_36_H_40_N_2_O_6_	NS	E	[38]
32	*N*-Norisoliensinine	C_36_H_40_N_2_O_6_	NS	E	[38]
33	Nelumborine	C_36_H_40_N_2_O_6_	NS	E	[45]
**TRIBENZYLISOQUINOLINE**					
34	Neoliensinine	C_63_H_70_N_3_O_10_	1*R*,1′*S*,1″*R*	E	[46]

**Table 2:** BIA biosynthetic enzyme candidates potentially involved in 1-benylisoquinoline, aporphine and bisbenzylisoquinoline pathways in sacred lotus. For each enzyme a proposed substrate(s) and product are shown. Accession numbers in ^a^ GenBank or ^b^ lotus databases (lotus-db.wbgcas.cn [1]) are provided. 4-HPAA: 4-hydroxyphenylacetaldehyde, 4′OMT: 4′-*O*-methyltransferase, 6OMT: norcoclaurine-6-*O*-methyltransferase, 7OMT: 7-*O*-methyltransferase, CNMT: coclaurine *N*-methyltransferase, CYP719A: cytochrome P450 monooxygenase 719A, CYP80A: cytochrome P450 monooxygenase 80A, CYP80G: cytochrome P450 monooxygenase 80G, NCS: norcoclaurine synthase.

Class	Enzyme	Isoforms	Substrate	Product	Reference
Pictet-Spenglerase	NCS	NCS1 (KT963033) ^a^	Dopamine 4-HPAA	Norcoclaurine	[34]
		NCS3 (KT963034) ^a^			
		NCS4 (KT963035) ^a^			
		NCS5 (KU234431) ^a^			
		NCS7 (KU234432) ^a^			
*O*-Methyltransferase	6OMT	6OMT1 (MG517493) ^a^	Norcoclaurine	Coclaurine	[49,61,62]
		6OMT2 (MG517492) ^a^			
		6OMT3 (MG517491) ^a^			
		6OMT4 (MG517490) ^a^			
	7OMT	7OMT1 (NNU20903) ^b^	Coclaurine	Norarmepavine	[61]
		7OMT2 (NNU04966) ^b^			
		7OMT3 (NNU09736) ^b^			
	4′OMT	4′OMT1 (NNU15801) ^b^	*N*-Methylcoclaurine	4′-*O*-methyl-*N*-methylcoclaurine	[49]
		4′OMT2 (NNU15809) ^b^			
		4′OMT3 (NNU24728) ^b^			
		4′OMT4 (NNU25948) ^b^			
*N*-Methyltransferase	CNMT	CNMT1 (MG517494) ^a^	Coclaurine	*N*-Methylcoclaurine	[49,61,62]
		CNMT3 (MG517495) ^a^			
Cytochrome P450 monooxygenase	CYP80A	CYP80A (NNU21373) ^b^	*N*-Methylcoclaurine	Nelumboferine	[61]
	CYP80G	CYP80G (NNU21372) ^b^	*N*-Methylcoclaurine	Lirinidine	[49,61,62]
	CYP719A	CYP719A22 (XM010268782) ^a^	Lirinidine	Roemerine	[61,69]
